# Extensive macular atrophy with pseudodrusen-like appearance: comprehensive review of the literature

**DOI:** 10.1007/s00417-024-06600-z

**Published:** 2024-08-12

**Authors:** Matteo Mario Carlà, Federico Giannuzzi, Francesco Boselli, Emanuele Crincoli, Stanislao Rizzo

**Affiliations:** 1 Ophthalmology Department, Fondazione Policlinico Universitario A. Gemelli, IRCCS, Rome, Italy; 2 Catholic University “Sacro Cuore”, Rome, Italy

**Keywords:** Extensive macular atrophy with pseudodrusen, Reticular pseudodrusen, Optical coherence tomography angiography, Complement cascade, Basal laminar deposits, Age-related macular degeneration, Multimodal imaging

## Abstract

**Purpose:**

This review focuses on extensive macular atrophy with pseudodrusen-like appearance (EMAP), a recently described maculopathy presenting with pseudodrusen-like lesions and chorioretinal atrophy more pronounced in the vertical axis.

**Methods:**

Narrative review of the literature published until May 2024.

**Results:**

The early onset age of EMAP (50–55 years) and its distinctive natural history, which includes night blindness followed by severe vision loss, differentiate it from atrophic age-related macular degeneration (AMD). A clear pathogenesis has not been determined, but risk factors include female gender and complement system abnormalities (altered levels of C3 and CH50). Moreover, lifelong exposure to pesticides has been suggested as risk factor for direct neuronal degeneration involving rods and cones. In the early phase of the disease, reticular pseudodrusen-like lesions appear in the superior perifovea and tend to coalescence horizontally into a flat, continuous, reflective material localized between the retinal pigmented epithelium and Bruch’s membrane. Over time, EMAP causes profound RPE and outer retinal atrophy in the macular area, with a recent classification reporting a 3-stages evolution pattern. Blue autofluorescence showed rapidly evolving atrophy with either hyperautofluorescent or isoautofluorescent borders. Significant similarities between the diffuse-trickling phenotype of geographic atrophy and EMAP have been reported. Macular neovascularization is a possible complication.

**Conclusion:**

EMAP is specific form of early-onset atrophic macular degeneration with rapid evolution and no treatment. Further studies are needed to assess the best management.

## Key messages

- ***What is known***
• Extensive Macular Atrophy with Pseudodrusen (EMAP) is a rapidly-progressive macular atrophy developing in the fifth-decade and leading to legal blindness.
• Typical characteristics at multimodal imaging are reticular pseudodrusen-like lesions starting at the superior perifovea and vertically-oriented macular atrophy.
- ***What is new***
• Dysregulated complement cascade and pesticide-related toxicity have been proposed as possible etiologies.
• In the late stages of the disease 50% of EMAP patients were legally blind by US standards and, after 7 years on average, atrophy reaches the mid-periphery.

## Introduction

The condition known as extensive macular atrophy with pseudodrusen-like appearance (EMAP) was first described by Hamel et al. in 2009. During an 18-year monitoring period, they identified 18 individuals who had “bilateral polycyclic well-delineated chorioretinal atrophy extending to the temporal vascular arcades, with a larger vertical axis and without sparing of the fovea” [1].

In this condition, the whole posterior pole and the midperiphery are lined with diffuse reticular pseudodrusen (RPD), evolving towards symmetric, bilateral widespread geographic atrophy affecting the posterior pole without foveal sparing [1]. In addition, paving stone lesions in the far peripheral retina are also observed in most EMAP patients [2].

Symptoms often start to exhibit in the fifth decade, with patients experiencing progressive night blindness, characterized by a fast, bilateral, and central reduction in vision [3, 4]. The first reports showed that two-third of patients had poor vision as early as four years after diagnosis [4]. Even though pseudodrusen lesions may be linked also to age-related macular degeneration (AMD), several clinical differences with EMAP have been reported: in the latter, the age at onset is much earlier, both eyes are affected simultaneously and the deterioration of central vision is faster [3].

At the final stages of EMAP, blue fundus autofluorescence (B-FAF) showed single patches of dark and sharply demarcated atrophy, while optical coherence tomography (OCT) revealed invisible photoreceptor cell line and macular thinning. The analysis of the first cases highlighted absolute central scotomas on Goldmann perimetry, anomalous color vision test findings (blue-yellow axis dyschromatopsia) and subnormal scotopic and photopic responses on the full-field electroretinogram (ERG) [5–9].

Since its discovery, a several publications have been released regarding this pathology, providing further details regarding the genesis, clinical trajectory, and multimodal analysis patterns. As far as we know, this is the first review gathering the state of the art on this condition.

## Methods

This is a narrative review conducted at the Ophthalmology Department of Fondazione Policlinico Universitario “A. Gemelli”, IRCCS. None of the writers performed any new investigations involving humans or animals; instead, this narrative review is solely based on earlier research, thus the Institutional Review Board approval was waived.

We searched the Cochrane Central, PubMed, Web of Science, and ClinicalTrials.gov databases for papers regarding EMAP, looking for the following keywords: “extensive macular atrophy with pseudodrusen”, “extensive macular atrophy with pseudodrusen-like appearance” and “EMAP”. Databases were accessed on May 2024. We only took into account English-language articles. None of the writers performed any new investigations involving humans or animals; instead, this narrative review is solely based on earlier research.

### Etiology and risk factors

There is currently no documented etiology or established pathogenesis for EMAP. Risk factors for EMAP may not be the same as those for traditional AMD [10, 11]. Specifically, even if previous studies focused on small sample sizes, no apparent correlation was found with cardiovascular risk factors, such as smoking status, body mass index, hypertension, diabetes, low-density lipoprotein (LDL) cholesterol, serum total and high-density lipoprotein (HDL) cholesterol, and triglyceride levels [3]. These findings suggest that the development of EMAP is not related to cardiovascular imbalance or the ensuing vascular insufficiency at the choroid level, being significantly different from pseudodrusen AMD, which has previously been associated with cardiac risks, cardiovascular illness, and choriocapillaris perfusion imbalance [11, 12]. 

Conversely, the role of immunological pathways, particularly complement cascade, was investigated. The EMAP Case-control National Clinical Trial evaluated 115 patients from various French centers and found risk factors for complement pathway dysfunction (abnormal levels of C3 and CH50), gender (women tended to be more affected, with rates ranging from 52.5 to 60.8%), and other abnormal levels of inflammatory markers (erythrocyte sedimentation rate, eosinophils, and lymphocytes), as well as a potential correlation with a family history of glaucoma and AMD [3]. These findings support the theory that an inflammatory state with an autoimmune disease background serves as the trigger for EMAP pathogenesis [7]. Moreover, the development of RPD may the consequence of this inflammatory state, since in AMD the presence of RPD has been claimed as a biomarker of the retinal pigmented epithelium (RPE) inability to control and limit the damage related to the loss of regulatory immune responses (para-inflammation) [13]. 

As a result, similar to AMD, inflammation might play a role in EMAP, with further research needed to assess if complement cascade activation is involved to a greater extent [3]. Genetic analysis of risk alleles including those of the alternative complement pathway, was performed in 65 EMAP patients. Heterozygous and homozygous variations for the rs1061170 (His402Tyr) and for the rs800292 (Val62Ile) in *CFH* gene were reported, with the first being more frequent (around 80% of cases). Five and 25 patients were homozygous and heterozygous for the rs10490924 (Ala69Ser) in *ARMS2* gene, respectively. For the rs2230199 (Arg102Gly) in C3, two patients were homozygous and 20 were heterozygous [2]. When comparing the rates of these variations with the genetic profile of classic AMD, no significant changes were reported regarding *CFH* and *ARMS2* genes. Conversely, in EMAP patients, the mutation rs2230199 (Arg102Gly) in C3 was substantially more common [2]. 

Previous research linked the dysregulation of the alternative complement pathway to dense deposit disease (DDD), characterized by electron-dense deposits in the glomerular basement membrane (GBM) of the kidney, and basal laminar deposits (BLamD) in Bruch’s membrane (BrM) [14]. This term refers to the presence aberrant basement membrane proteins embedded in long-spacing collagen, situated between the natural basal lamina (BL) and the RPE plasma membrane [15]. Boon et al. reported the presence of BLamD in a variant of AMD with Tyr402His mutation in the *CFH* gene, featured by early onset and severe vision loss [16]. Similarly, Fragiotta et al. recently reported the presence of BLamD, located in the superior macula, in the very early stages of EMAP, suggesting a common pathway of immune dysregulation [15]. 

As of now, neither a definite genetic correlation, nor a familial transmission of the disease, have been demonstrated yet. Some cases of genetic correlation were recently reported, but a clear association with EMAP pathogenesis has yet to be defined [17, 18]. 

Douillard et al. recently carried out a second investigation with the same group of the EMAP Case-control National Clinical Trial, suggesting a direct neuronal degeneration involving rods and cones and identified a correlation between lifetime toxic exposure (pesticides used in agricultural operations) and EMAP [2]. Even though the precise molecular processes of neuronal degeneration are yet unknown, previous research highlighted that the majority of pesticides cause changes in the mitochondria, oxidative or endoplasmic reticulum stress, and ultimately death of neuronal cells [19]. Consequently, the authors suggested a possible parallelism between EMAP and other toxic-induced neuronal degeneration, such as Parkinson’s disease [2]. EMAP-induced neuronal degeneration may affect either rods and cones, but electrophysiology studies suggest a predominant cone apoptosis [2]. 

Even if only preliminary theories have been proposed, EMAP pathogenesis seems to be the result of a lifelong exposure to external toxic agents determining a state of subclinical chronic inflammation, which acts as a trigger in a substratum of pre-existing abnormal complement pathway regulation. Further studies are needed to give light to this hypothesis.

### Clinical course and prognosis

Since EMAP is a recently defined condition, a precise clinical course has not been defined, yet. Visual symptoms usually begin between 48 and 60 years of age, with a mean age of diagnosis of 55 years in a recent investigation [4]. Initially, patients refer difficulties with dark adaptation, which rapidly progress to the development of central scotomas. Inevitably, visual acuity deteriorates to legal blindness (vision of 20/200 or less) in 3 to 10 years from diagnosis [1]. In two studies analyzing visual outcomes in EMAP patients, best-corrected visual acuity (BCVA) declined linearly from baseline to the last visit, with a rate of around 7 Early Treatment Diabetic Retinopathy Study (ETDRS) letters lost per year [4, 20]. Overall, nearly 65% of eyes lost 3 or more ETDRS lines after 4 years of follow-up, with a BCVA of 35 ETDRS or less in 57% of eyes [4]. 

Nevertheless, the progression rate of BCVA, similar to that of RPE atrophy, showed significant interpatient variability, highlighting the need for further longitudinal studies [4]. 

### Optical coherence tomography

Reticular pseudodrusen, also known as subretinal drusenoid deposits (SDDs), have been defined as well-defined conical (“dots”) or flat (“ribbon”) hyperreflective deposits among the RPE and the boundary between photoreceptor inner and outer segments [21]. In EMAP, several authors claimed the presence of RPD-like lesions as significant hallmark of the pathology.

In a group of EMAP patients ranging from 45 to 53 years, Fragiotta et al. reported the presence of RPD/SDD-like deposits without atrophic changes [15]. On structural OCT, SDD appeared in the superior perifovea and tended to coalescence horizontally into a flat, continuous, reflective material localized between the RPE and BrM, which determined a diffusely irregular RPE profile. The interdigitation zone (IZ) and ellipsoid zone (EZ) showed, in this area, a scattered granular hyperreflectivity [15]. Following a 4-year period of observation, an hyperreflective material had accumulated under the dysmorphic RPE and EZ/IZ lines were no more visible. The presence of this medium-reflectivity interposed substance determined a clear RPE–BL–BrM complex splitting with a noticeable BrM. At this point, complete RPE and outer retinal atrophy (cRORA) occurred, starting from the superior perifovea [15]. 

**Fig. 1:**
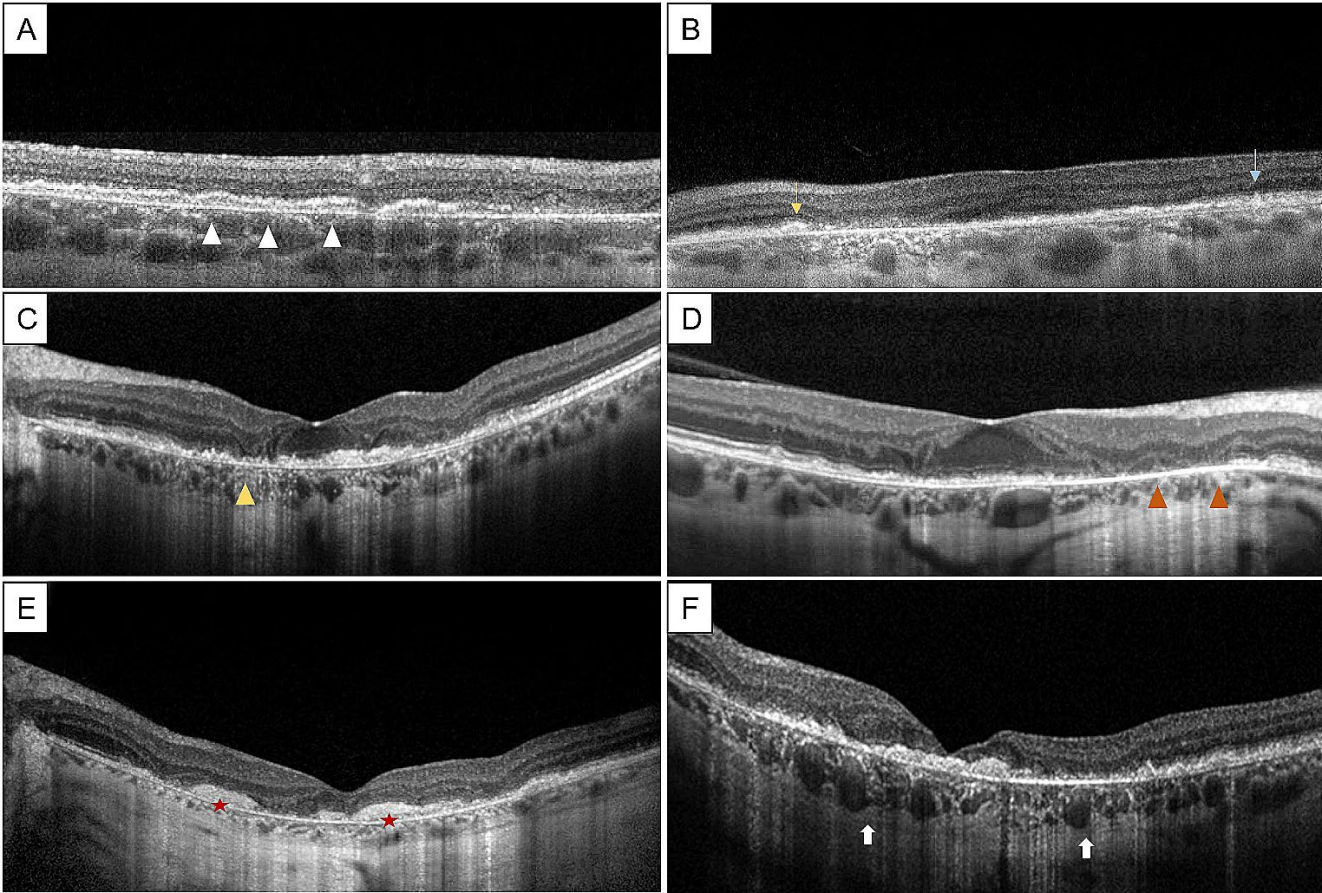
Optical coherence tomography B-scans in extensive macular atrophy with pseudodrusen (EMAP), at different stages of evolution. **A** Early findings show an initial retinal pigmented epithelium-basal lamina-Bruch’s membrane (RPE-BL-BrM) complex splitting (*white arrowheads*) with an overlying dysmorphic and irregular RPE in the superior perifovea. **B** Subretinal deposits (SDDs) may show a “ribbon” shape (*yellow arrow*) or a “dot” shape (*turquoise arrow*). **C** In intermediate stages, RPE-BL-BrM splitting is visible in the foveal area, with hyperreflective material under the RPE. Moreover, a localized area of complete RPE and outer retinal atrophy (cRORA) is visible (*yellow arrowhead*); **D** Macular scan showing collapse of the nasal perifoveal outer retinal layers with choroidal hypertransmission (*yellow arrowheads*), while the fovea is still unaffected and shows RPE irregularities; **E** In late stages of the disease, cRORA involves the foveal area and subretinal fibrosis may develop (*red stars*). **F** Complete choroidal hypertransmission with clear visibility of choroidal vessels (*white arrows*) due to complete RPE atrophy. Courtesy *of Fragiotta et al.* [15] *Antropoli et al.* [41] *and Romano et al.* [4]

Similarly, Romano et al. reported a noticeable separation of the RPE from the BrM and thinning of the outer nuclear layer (ONL) on OCT, followed by the breakdown of the hyperreflective outer retinal bands, as the earliest findings of EMAP [20]. Among 17 eyes, RPE-BrM separation was already observed in all patients at presentation, when considering the extrafoveal macular areas, and in the majority of eyes (88.2%) at the foveal area. All eyes had overall thinning of the ONL, and 58.8% showed diffuse irregularity of the EZ, which became disrupted in seven eyes (41.2%) throughout the follow-up. In all instances, EZ disruption occurred prior to external limiting membrane (ELM) changes, and appeared in concomitance with RPE layer attenuation [20]. In the end, cRORA progressively developed with a typical barcode appearance, leaving space to persistent subretinal heterogeneous hyperreflective material and to subsidence of the outer plexiform layer (OPL). After an average of 17.5 months of follow-up, subfoveal fibrosis in the absence of intraretinal/subretinal fluid was seen in 11 eyes, starting in the perifoveal area and then spreading to the fovea [20] (Fig. 1).

At the ophthalmoscopic examination, EMAP’s atrophy assumes a predominant vertical orientation at the posterior pole. This feature derives from RPE changes primarily affecting superior perifoveal sector and then spreading towards the fovea. Pseudodrusen-like lesions and the early cRORA patches have a preferred superior localization probably due to the larger density of rod photoreceptors in those places as well as the lower perfusion caused by gravity [22, 23]. Unlike AMD, the majority of these RPD lesions have a “ribbon” appearance and uniformly extend to the retinal midperiphery rather than being restricted to the posterior pole. Conversely, “dot” pseudodrusen-like lesions are more uncommon in EMAP patients and are restricted to the macular area, similar to AMD [24]. When macular atrophy appears, it shows an oval shape progressively evolving from the superior macula to the other Early Treatment Diabetic Retinopathy Study (ETDRS) sectors [20]. 

### Optical coherence tomography angiography

Only few reports have been presented regarding the application of OCT angiography (OCTA) technology in EMAP patients. Kovach was the first to analyze OCTA findings in a patient affected by EMAP, reporting marked absence of choriocapillaris (CC) flow and mild attenuation of retinal vasculature [5]. 

Successively, Rajabian et al. performed an OCTA investigation comparing 14 eyes with EMAP with healthy controls. With respect to the progression of the disease, they divided the macular area in an atrophic area, a junctional area (which became atrophic during the follow-up) and a preserved area (non-atrophic) [6]. OCTA showed an almost preserved superficial capillary plexus (SCP) in all zones, when compared with controls. Conversely, they showed a reduction of vessel density in the deep capillary plexus (DCP) in either the junctional and atrophic zones, but not in the preserved zone, suggesting that DCP changes appear earlier in EMAP [6]. Interestingly, CC vascular architecture showed progressive damage when going from the preserved retina (highest vessel density), to the junctional region (intermediate vessel density) and to the atrophic region (lowest vessel density), with statistically significant differences between the three regions [6]. In conclusion, the authors hypothesized that CC alterations may occur more slowly in EMAP physiophatology, rather than DCP alterations which were most clearly identifiable in the junctional area [12]. 

### Fundus autofluorescence

**Fig. 2:**
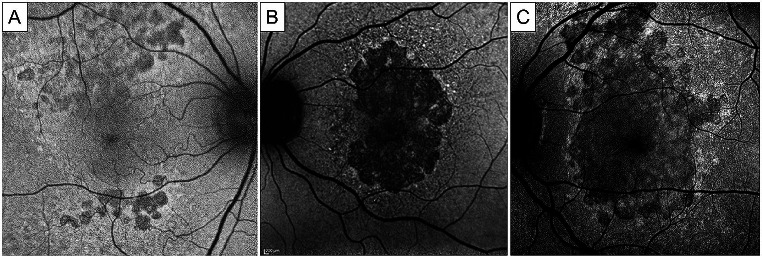
Blue fundus autofluorescence photographs of extensive macular atrophy with pseudodrusen (EMAP), showing the classic evolution pattern. Hypoautofluorescence lobular lesions are visible in perifoveal areas, particularly the superior perifovea (**A**). These multifocal lesions tend to coalesce into a single patch of dark and sharply demarcated atrophy, initially not affecting the fovea (**B**). In advanced stages, a vertically oriented area of atrophy involves the macular region and involves the fovea (**C**). Note the hyperautofluorescent rim visible at the boundaries of the atrophic lesion. *Courtesy of Antropoli et al.* [41], *Romano et al.* [4] *and Vilela et al.* [9]

Blue-light fundus autofluorescence (BAF) has been used to assess the progression of macular atrophy in EMAP. In a study carried out by Romano et al. on 36 eyes, BAF showed signs of RPE atrophy at the posterior pole in all instances, with 72.2% of eyes demonstrating hyperautofluorescent borders, whereas the remaining 27.8% showed iso-autofluorescent borders [4]. They reported that the advancement rate in EMAP is greatly impacted by the identification of iso-autofluorescent and more irregular atrophy boundaries. While the irregular atrophy border structure has been associated with a quicker rate of atrophy development in other maculopathies, the autofluorescence patterns found in the junctional area appeared different from classic geographic atrophy (GA) [4]. However, although the identification of hyper-autofluorescent borders was initially linked to rapidly developing forms of GA, recent clinicopathologic investigations detected that the hyper-autofluorescent rim is probably caused by stacked RPE cells and not associated with atrophy enlargement rate [25, 26]. Romano et al. hypothesized that atrophy will most likely enlarge in locations where RPD deposits have coalesced, and that the visible iso-autofluorescent pattern is the result of an heterogeneous material composition in the junctional area [4] (Fig. 2).

The annual rate of atrophy expansion, evaluated with BAF, was 2.9 mm^2^/year, leading to foveal involvement in 83% of eyes at the end of follow-up. This progression rate was higher than AMD-related GA progression (range 0.53–2.60 mm^2^/year), but more similar to the diffuse-trickling geographic atrophy (DTGA) subtype [27]. 

Fragiotta et al. reported, in the early phases of the pathology, a diffusely reduced BAF signal in the foveal region corresponding to RPE rarefaction with a remarkable RPE-BrM splitting and sub-RPE basal laminar deposits (BLamD) accumulation [15]. This reduced signal may be the effect of an altered metabolic trafficking between choroid and RPE, leading to a local deficiency of vitamin A, or may be caused by the loss or rearrangement of autofluorescent organelles from individual RPE cells [28, 29]. Furthermore, a fine granular hyperautofluorescence was noted at the boundaries of the atrophic lesion, probably originating from the staining of the sub-RPE material [15] [Figure 1].

Rabinovich et al. exploited the the semi-automated RegionFinder™ software (Heidelberg, Germany) to assess the development of atrophy in eyes with EMAP, compared to eyes with a subtype of Stargardt disease, known as fundus flavimaculatus (FFM): the first appeared to be about six times quicker than the latter (3.73 vs. 0.70 mm^2^/year) [8]. Atrophic expansion linked to both FFM and EMAP was non-linear over time, since the progression rate slowed down after an average of 1.3–1.6 years [8]. 

Recently, Battaglia Parodi et al. described peripheral retina alterations in EMAP patients using ultrawidefield fundus photography (UWFFP) and ultrawidefield fundus autofluorescence (UWF-FAF). They identified three main types of peripheral degeneration: RPE alterations, pavingstone-like changes, and pigmented chorioretinal atrophy. These degenerations progressed in two-third of eyes over time, at a median rate of 0.7 sectors/year. Moreover, enlarging rate of macular atrophy, after the square root transformation, was 0.46 ± 0.28 mm/year [30]. 

### EMAP stages

A recent research proposed a three-stage EMAP categorization scheme, based on multimodal imaging results and visual symptoms [20] (Table 1). Moreover, a recent report distinguished EMAP pattern based on topographic involvement: predominantly central, predominantly peripheral and mixed forms [30]. 

**Table 1:** Clinical staging of EMAP as proposed by Romano et al., with the pre-EMAP stage proposed by Fragiotta et al.

Stages	Multimodal imaging	Symptoms
Pre-EMAP	Presence of RPD in the superior perifovea, leaving place to BLamD; intact RPE	None
1	Widespread RPE–BrM separation with focal or diffuse EZ attenuation; absent or minimal patches of RPE atrophy	Impaired dark adaptation and altered color perception
2	Appearance of several patches of RPE atrophy (predominantly in superior sector) but fovea is still spared	Impaired dark adaptation, near vision difficulties, mild visual loss
3	Extensive macular atrophy with foveal involvement, with two different aspects: A) Foveal atrophy B) Subfoveal fibrosis	Severe visual loss and significant impact on quality of life. Ultimately, legal blindness
+	MNV development	Metamorphopsias and/or sudden visual loss

*EMAP* extensive macular atrophy with pseudodrusen, *RPD* reticular pseudodrusen, *BLamD* basal laminar deposits, *RPE* retinal pigmented epithelium, *BrM* Bruch’s membrane, *EZ* ellipsoid zone, *MNV* macular neovascularization

**Fig. 3:**
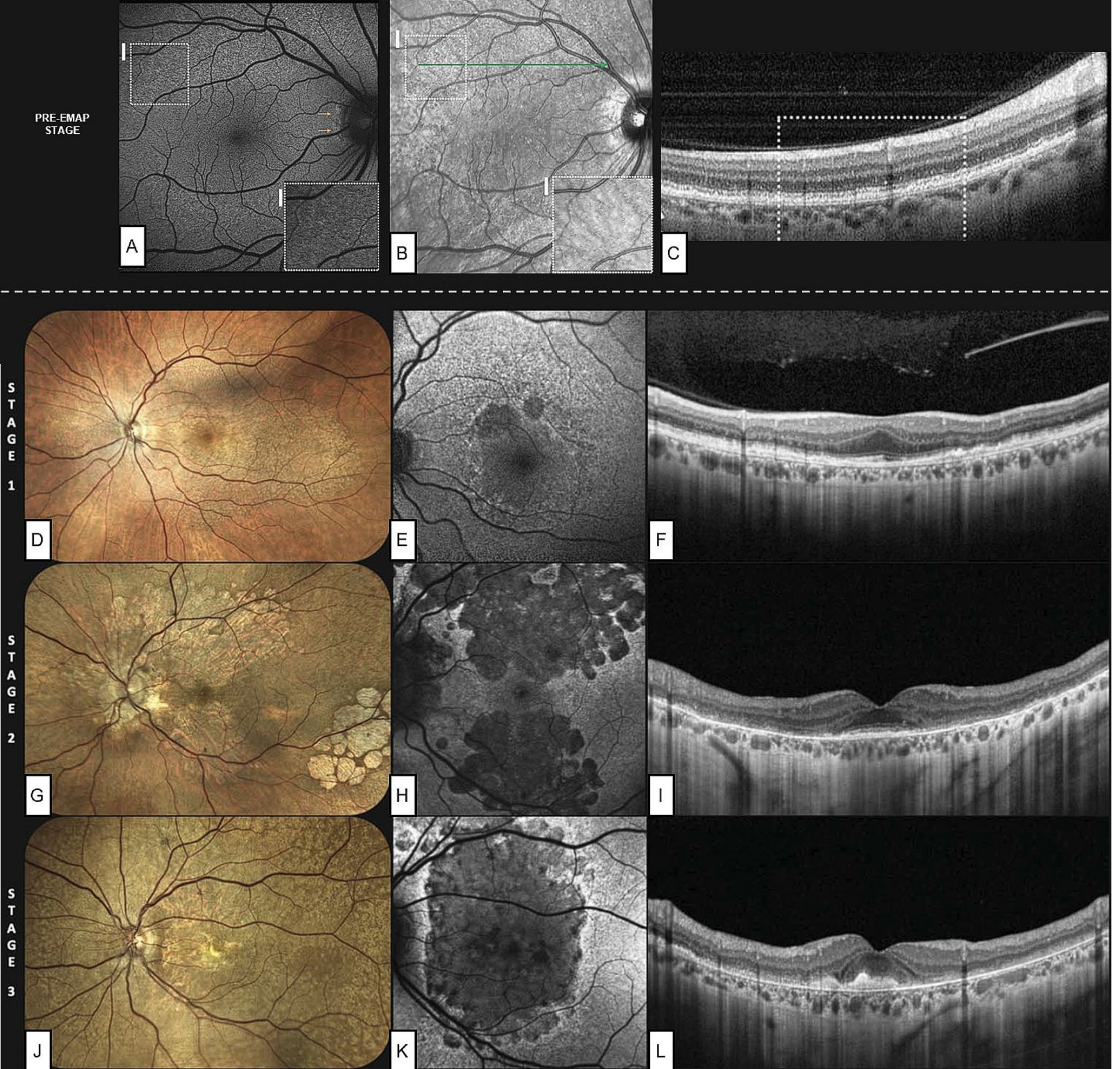
Staging system proposed by Romano et al., with the addition of the pre-extensive macular atrophy with pseudodrusen (pre-EMAP) stage, proposed by Fragiotta et al. Mild autofluorescence (**A**) and infrared (**B**) alterations are visible in the superior perifovea, corresponding to RPE granularity (**C**) and initial splitting of the retinal pigmented epithelium-basal lamina-Bruch’s membrane (RPE-BL-BrM) complex. Fundus photographies (**D, G, J**), fundus autofluorescence (**E, H, K**) and optical coherence tomography scans passing through the fovea (**F, I, L**), show the progressive atrophy involving the posterior pole and assuming a vertically-oriented shape. Courtesy *of Fragiotta et al.* [15] *and Romano et al.* [20]

Stage 1 is characterized by the splitting of RPE–BrM and scattered RPDs extending into the retinal midperiphery, with non-confluent patches of RPE atrophy limited to the superior macula. In this stage, a good preservation of visual acuity is maintained. In Stage 2, RPE atrophy appears confluent and widespread, acquiring a prominent vertical direction, but the fovea is still preserved. Though visual acuity stays comparatively high, visual symptoms such as reading difficulties and poor dark adaption become more noticeable. In Stage 3, the illness affects the fovea, with a consequent significant decline in visual acuity. Moreover, foveal involvement may result from either RPE atrophy (stage 3 A) or subretinal fibrosis development (stage 3B) [20] (Fig. 3).

**Fig. 4:**
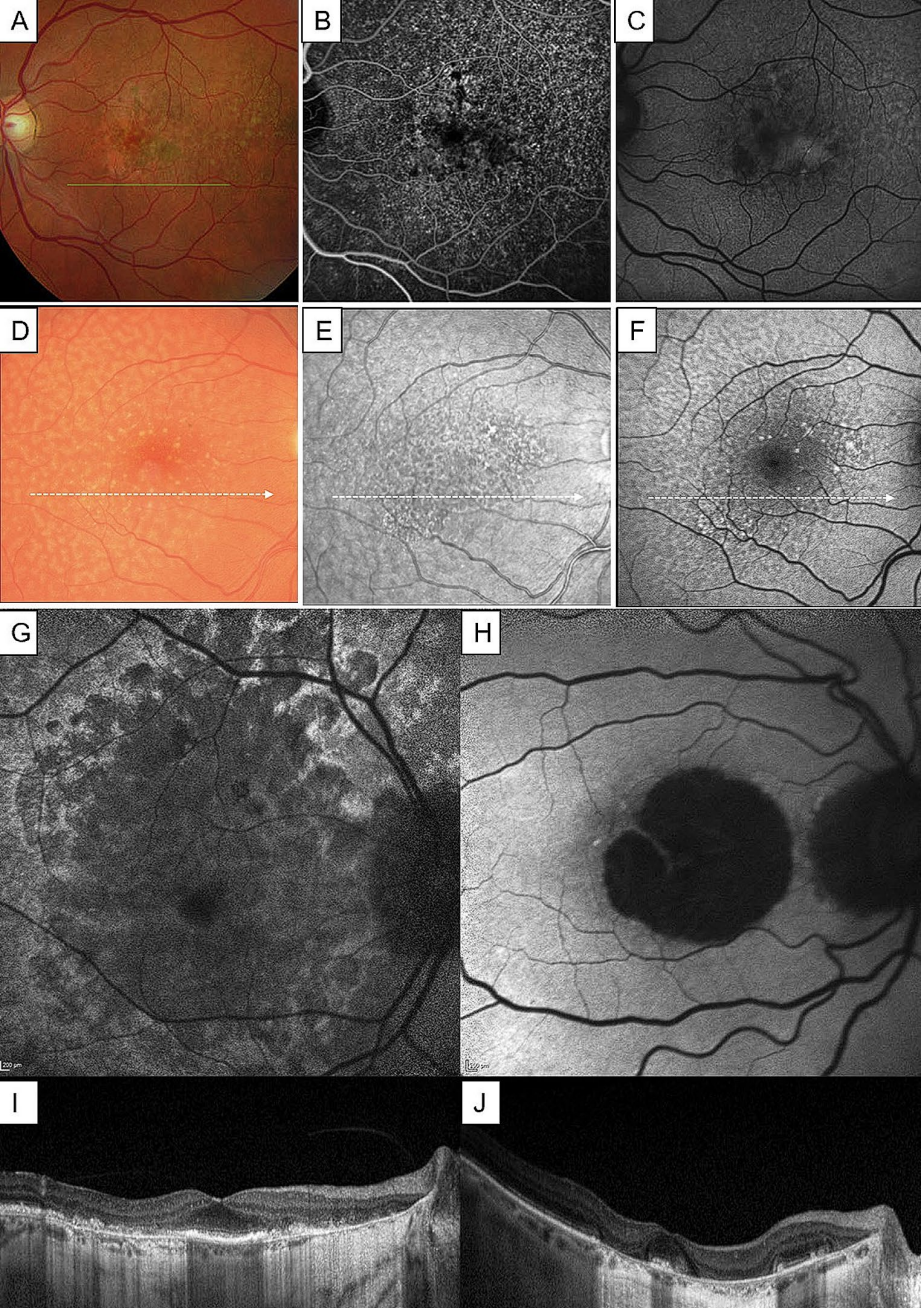
Multimodal imaging of differential diagnosis of extensive macular atrophy with pseudodrusen (EMAP). Cuticular drusen, seen at the fundus photography (**A**) and showing the “stars in the sky” appearance at fluorescein angiography (**B**), with corresponding punctate hypo-autofluorescence areas at the autofluorescence (**C**). Reticular pseudodrusen, seen at fundus photography (**C**), infrared image (**E**) and autofluorescence (**F**), tend to coalesce and cause central atrophy, but typical onset is > 65 years of age. Differences between autofluorescence atrophy patterns of diffuse-trickling phenotype of geographic atrophy (DTGA, **G**), which is much more similar to EMAP, when compared to classic geographic atrophy (GA, **H**), showing a central area of hypoautofluorescence with a major horizontal axis. Correspoding optical coherence tomography images of DTGA (**I**) and GA (**J**). *Courtesy of Sakurada et al.* [38], *Wu et al.* [40] *and Antropoli et al.* [41]

**Fig. 5:**
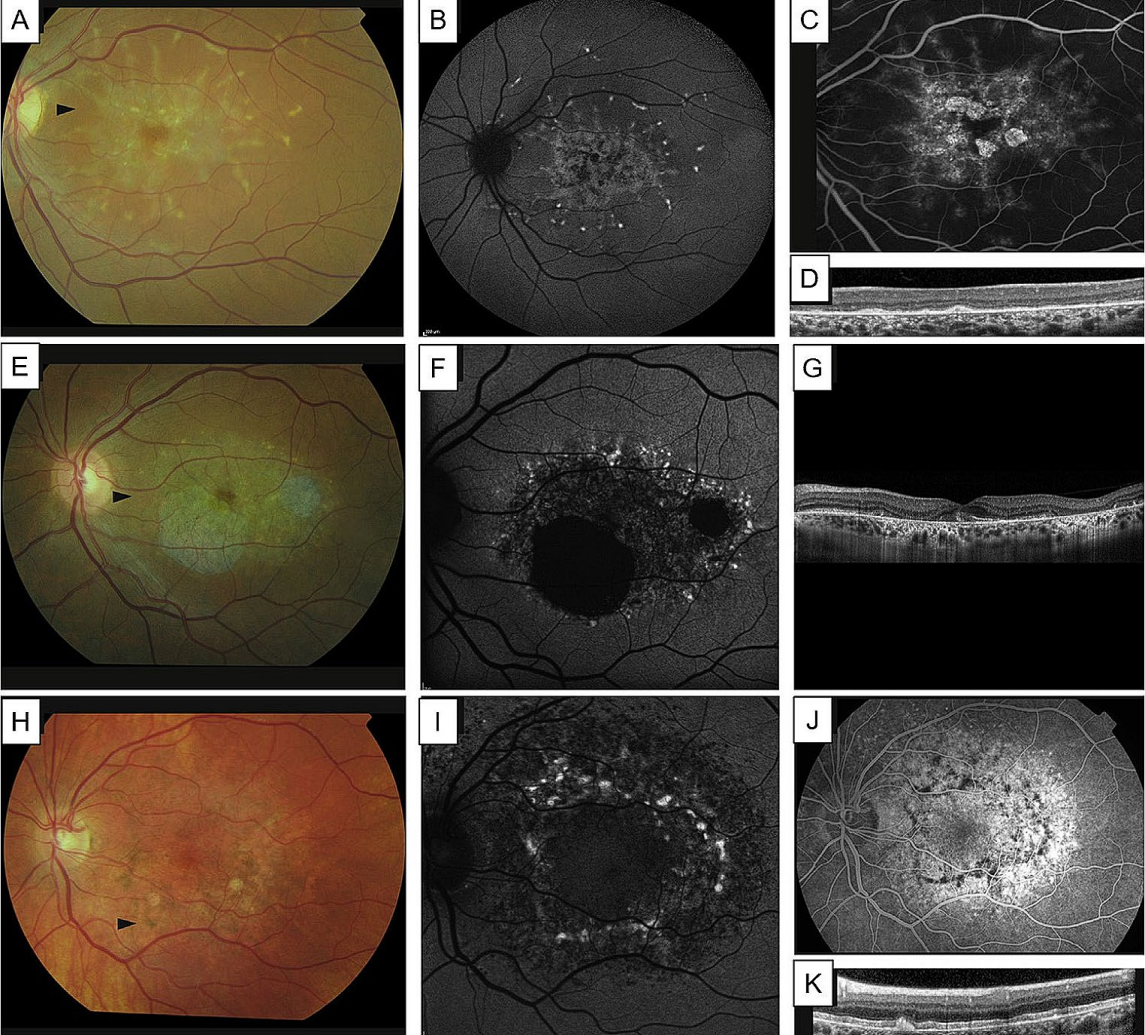
Multimodal imaging of differential diagnosis of extensive macular atrophy with pseudodrusen (EMAP) with other retinal dystrophies. (**A-D**) Late-onset Stargardt disease, showing flavimaculatus flecks scattered throughout the posterior pole (**A, B**), the presence of a “dark choroid” at fluorescein angiography (**C**) and hyperreflective thickening of retinal pigmented epithelium (RPE) layer at the optical coherence tomography (OCT) scan (**D**). (**E-G**) Central areolar choroidal dystrophy linked to *PRPH2* mutation, showing hypopigmentation of the macula with oval-to-round, atrophic areas (**A**) and corresponding areas of severely decreased to absent autofluorescence with perilesional spots of hyperautofluorescence (**F**). (**G**) Horizontal OCT scan showing well circumscribed chorioretinal atrophy. (**H-K**) m.3243 A > G Macular dystrophy, visible as hyperpigmented irregular spots in the macula and an oval area of mottled hypopigmentation at fundus photography (**A**). Fundus autofluorescence (FAF) image showing that ovally-shaped hyperautofluorescent areas, corresponding to hyperpigmented spots, and diffuse irregular hypoautofluorescence (**I**). Fluorescein angiography (FA) showing blockage of background fluorescence in the area of hyperpigmentation and a diffuse hyperfluorescence due to a RPE window defect (**J**). Hyperreflective spots originates from dome-shaped hyperreflective lesions of the RPE visible at OCT scans (**K**). *Courtesy of Saksens et al.* [39]

Fragiotta et al. highlighted a very early stage of the disease, featuring the presence of BLamD. This appearance is characterized by a hyperreflective intact RPE band, interposed with a thin hyporeflective band that makes the underneath hyperreflective BrM visible and thickened [31]. According to Fragiotta and colleagues, this pre-EMAP stage is defined by the presence of RPD/SSD-like deposits in the superior perifovea and midperiphery, that mostly affect young patients. These deposits are gradually replaced by BLamD, causing RPE-BrM complex splitting, but showing an intact RPE layer [15]. 

Finally, the late stages of the disease were recently explored in a worldwide cohort of 156 eyes: at a mean age of 70 years old, 50% of EMAP patients were legally blind by US standards, even if only 25% of them satisfied the World Health Organization’s (WHO) < 20/400 blindness threshold. On the other hand, a vision of 20/200 or worse is usually reached after around 4 years, in line with the median amount of time of foveal atrophy development. After an average of 4.7 years, the atrophy moves beyond the central 30°, and after 7 years or more, it reaches the mid-periphery [32].

### Electrophysiology

In the first report by Hamel et al., full-field electroretinogram (ERG) on EMAP patients showed dark-adapted scotopic subnormal responses, compatible with rod dysfunction, which may be caused by RPD-like lesions. Moreover, photopic responses were likewise subnormal, probably due to atrophy-related cone degeneration [1]. Moreover, the distribution of cone density, raising from a 7 mm (35°) eccentricity line (20.000 cones/mm^2^) to the fovea peak cone (150.000 cones/mm^2^), reflects the pattern and severity of 25–35° central scotomas in EMAP [33]. In the investigation of Douillard et al., EMAP patients, differently from AMD, showed severe decline in photopic single flash and flicker ERG responses, suggesting early and severe cone degeneration [2]. 

In a novel research by Watanabe et al., 18 EMAP patients underwent full-field ERG (ffERG), showing altered scotopic and photopic conditions in 78% of cases [7]. Macular response, evaluated by multifocal ERG (mfERG) in 11 patients, showed decreased values in the 10 degrees of retinal eccentricity in all instances. Finally, nearly all of the patients (94%) showed ganglion cells dysfunction, when analyzed with the photopic negative response (PhNR) extended ERG protocol [7]. 

### Microperimetry

Romano et al. recently presented a study on 44 EMAP patients evaluated with microperimetry, compared to healthy subjects. Mesopic and scotopic retinal sensitivities were considerably lower, especially in the macular region. In particular, the early stage of the pathology linked with a reduction in scotopic sensitivity, suggesting an initial predominant rod impairment, caused by a defective metabolic transportation across the RPE-BrM complex [34]. Conversely, in more advanced EMAP stages, as far as cone dysfunction worsened, photopic retinal sensitivity gradually declined. Lower photopic sensitivity was linked to bigger atrophic regions, foveal involvement and fibrosis on multivariate analysis [34]. 

### Differential diagnosis

The main differential diagnosis of EMAP is atrophic AMD (dry AMD, dAMD). Differently from EMAP, GA in atrophic AMD develops after the age of 50, with atrophic lesions being the results of the convergence of drusen regression in many tiny foci [35, 36]. The progression of the atrophy from the perifovea to the fovea has a slow growth rate, resulting in a fovea-centered atrophic lesion with larger horizontal axis [36]. Moreover, classic GA is less widespread (not greater than 2 to 4 disc regions) when compared to EMAP atrophy [1]. A recent research showed that a deep learning classifier, based on FAF imaging, was able to distinguish EMAP from AMD on 30° × 30° images with a sensitivity of 85% and a specificity of 85%, and on 55° × 55° images with a sensitivity of 90% and a specificity of 85%. The use of wide field photos enabled a much increased sensitivity of the deep learning classifier, indicating the recognition of peripheral changes in the FAF pattern (such as pavingstone degeneration or RPE alterations), which characterize EMAP but not AMD [37]. 

Further, it is important to differentiate between EMAP, AMD with basal laminar drusen (cuticular drusen) and AMD with RPD. Cuticular drusen (CD) appear as slight thickening of the RPE/BrM complex (‘sawtooth’ RPE elevation) and show a specific FA pattern, called ‘stars-in-the-sky’, due to their hyperfluorescence. Moreover, in FAF, CD appear as punctate hypo-autofluorescence areas. The CD subtype of AMD showed strong association with variants in the *CFH* gene and an earlier age of onset. Typically, CD can cause visual loss as a result of vitelliform macular detachment or can coalesce and evolve towards GA, particularly the diffuse type. However, the OCT, FA and FAF aspects and the rate of progression are different from EMAP [38, 39]. 

On the other hand, RPD is associated with late AMD, showing higher correlation with GA and type 3 MNV. However, evidence for RPD as an independent risk factor for late AMD development is still debated. Typically, RPD are predominantly localized in the superior perifovea, show a progressive thickening over time and eventually fade. The presence of RPD has significant association with cardiovascular risk and environmental factors (such as smoking). Moreover, variations in the *CFH* gene suggest a complementary imbalance of the immune system at the basis of sub-retinal deposits development, similar to the hypothesized EMAP pathogenesis. Nevertheless, RPD in AMD are typically diagnosed in patients > 65 years, and the rate of progression in slower when compared to EMAP [40]. 

Interestingly, on the other hand, multimodal imaging showed several analogies between EMAP and the diffuse-trickling phenotype of geographic atrophy (DTGA). In fact, DTGA featured the same RPE-BrM splitting on OCT images, the grayish appearance of the atrophic lesions on autofluorescence, and the fast progression of macular atrophy with a major vertical orientation [27]. In order to assess this similarity, Antropoli et al. included in their study 18 patients with EMAP, 18 with DTGA and 27 with non-DTGA (nDTGA). They discovered that atrophy grows more quickly in EMAP and DTGA than in nDTGA, but the atrophic area at baseline was bigger in EMAP than in DTGA and nDTGA, probably due to patients’ underestimation of their symptoms in EMAP. Moreover, the atrophic area in EMAP had a lower circularity index than in nDTGA, but not compared with DTGA [41]. Bianco et al. extended the aforementioned investigation in a prospective study of 28 EMAP, 27 DTGA, and 30 non-DTGA eyes, confirming that EMAP is characterized by an irregular and elongated shape and has a fast atrophy growth rate (3.6 mm^2^/year), compared with non-DTGA. No differences were found between EMAP and DTGA in terms of progression rates and FAF aspect [42] (Fig. 4).

Choroidal insufficiency has been proposed by Fleckenstein et al. as possible pathogenetic mechanism of either DTGA and EMAP [27]. Choroidal vascularity index was indeed analyzed in the investigation of Antropoli and colleagues, but non-significant differences were reported between EMAP, DTGA and nDTGA [41]. Conversely, they detected choroidal caverns, defined as non-reflective spherical to polyhedral structures with a posterior hypertransmission tail on structural OCT, only in EMAP and DTGA; [43] notably, their prevalence increased over the course of the follow-up. The histological and physiological nature of choroidal caverns has not been clarified, with some authors considering them as “ghost vessels” [44]. 

The outer retinal layers are diffusely involved in both EMAP and DTGA, and the RPE-BrM splitting in visible in both conditions even far from atrophic areas, differently from nDTGA, in which RPE-BrM splitting is not always present. In summary, EMAP and DTGA are recognized among the most severe types of macular atrophy in terms of expansion and decline in visual acuity, showing also a number of common characteristics, which may indicate a shared pathogenetic origin [41]. 

EMAP should also be differentiated from late-onset retinal degeneration (L-ORD), sharing the same dark adaptation difficulty at 50 years old, the quick development of central vision loss, and widespread GA [45]. Unlike L-ORD, which is inherited as an autosomal dominant disease, EMAP did not show any signs of family heritability. Furthermore, the EMAP fundus pattern can be clearly differentiated from L-ORD, which is characterized by many atrophic patches that first affect the midperiphery [1]. 

Further differential diagnosis could be Sorsby fundus dystrophy (SFD), which also manifests with early bilateral vision loss, nyctalopia, and delayed dark adaption. However, SFD is entirely penetrant, autosomal dominant with varied expressivity, caused by mutations in the Tissue Inhibitor of Metalloproteinase-3 (TIMP3) gene. The typical result of SFD is central macular atrophy or scarring with the presence of macular neovascularization, leaving significant pigmentation after resolution [46]. 

Moreover, also the mitochondrial retinal dystrophy associated with the m.3243 A > G mutation has to be distinguished from EMAP. This condition is associated with a wide spectrum of retinal abnormalities, from pigmentary changes to macular atrophy. Hyperautofluorescent spots/flecks are visible in FAF, usually spreading along the temporal vascular arcades and around the optic disk, associated with areas of decreased FAF signal in correspondence to mild atrophy. OCT examination shows thickening of the EZ with RPE atrophy, evolving to the loss of outer retinal layers. The association with hearing loss, early onset diabetes and the maternal inheritance are key features for differential diagnosis with EMAP [39]. 

Central areolar choroidal dystrophy (CACD) is an autosomal dominant disorder caused by PRPH2 gene mutations, associated with visual deterioration in the fifth decade of life. This condition is associated with oval-to-round hypopigmented areas, visible as demarcated areas of FAF decreased signal, which tend to enlarge concentrically and become confluent. A disrupted EZ and reduced retinal thickness is visible on OCT. The shape of the atrophy and genetic test are the main differences with EMAP [39]. 

Finally, EMAP must be distinguished from late-onset Stargardt disease, which presents at the age of 55 with progressive central vision loss and irregular, pisciform, flavimaculatus flecks scattered throughout the posterior pole. Moreover, OCT shows hyperreflective thickening of RPE layer. However, the presence of a “dark choroid” at FA and the mutation of ABCA4 gene are hallmarks of Stargardt’s disease and not linked to EMAP [39] (Fig. 5).

### Complications

Some reports highlighted that macular neovascularization (MNV) is a potential complication of EMAP [47]. In particular, MNV seems to develop early and showed poor response to anti-vascular endothelial growth factor (anti-VEGF) injections, in the first reports [18]. 

Romano D et al. described spontaneous rupture of BrM and subsequent onset of MNV in an EMAP patient. They hypothesized that BLamD, characterized by high lipid content [31], may oxidate and set off an aberrant inflammatory response. Authors supposed that this hyperreflective material, visible in the RPE-BrM complex, consists of swollen, inflammation-activated cells, such as microglial cells and macrophages, with altered morphology, that tend to aggregate [48]. These activated cells express collagenase, elastase, nitric oxide synthase and other proangiogenic enzymes with proangiogenic, leading to BrM lesions and consequent CNV [49]. They further hypothesized that BrM disruption may be connected to the chronic inflammation and aberrant complement system regulation seen in EMAP [49]. As an alternative, they speculated that BrM rupture may be caused by worsening choroidal thinning, as seen in high myopia patients with lacquer crack lesions [49]. 

## Conclusions

In conclusion, EMAP is a recently described retinal condition causing early symptoms and fast progressing macular atrophy, inevitably leading to legal blindness, without an available effective treatment. First reports suggested a possible role inflammatory imbalance, as reported by abnormal C3 and CH50 levels. On the other side, the potential role of pesticide-related toxicity have been explored, possibly causing progressive neurodegeneration, affecting either rods and cones. Phenotypically, some analogies were reported with the DTGA subtype.

Further studies with larger cohorts and longitudinal follow-ups are needed, along with more insights into the pathogenesis, in order to expand our ability to discriminate different macular atrophy phenotypes and to optimize patient management. The authors hope that new research based on gene sequencing, adaptive optics or molecular analysis could be helpful to elucidate the pathogenetic mechanism underlying EMAP. Moreover, the application of artificial intelligence to OCT/OCTA and FAF findings could improve our capacity for predictive diagnosis and better management of this disease.

## Data Availability

The data that support the findings of this study are available from the corresponding author, MMC, upon reasonable request.
